# Autonomy and health-related quality of life in adolescents

**DOI:** 10.1186/s12887-022-03607-5

**Published:** 2022-09-20

**Authors:** Mårten Eriksson, Eva Boman, Pia Svedberg

**Affiliations:** 1 Faculty of Health and Occupational Studies, Department of Occupational Health Science and Psychology, University of Gävle, Gävle, Sweden; 2 Division of Insurance Medicine, Department of Clinical Neuroscience, Karolinska Institutet, Stockholm, Sweden

**Keywords:** Adolescents, Autonomy, Health-related quality of life, KIDSCREEN

## Abstract

**Background:**

Autonomy is recognized as important for individual well-being and constitutes one dimension in the KIDSCREEN-instrument measuring health related quality of life (HRQoL) in children and adolescents. However, the autonomy questions in KIDSCREEN are restricted to opportunities to influence leisure time activities, which is a form of autonomy as volition. Yet, there are other aspects of autonomy that might be related to adolescent’s HRQoL. The aims of the present study were first to investigate the psychometric properties of a scale measuring autonomy in adolescence from a control perspective (AAC) including its relation to the autonomy dimension in KIDSCREEN, and second; to investigate AACs ability to predict each of the 10 dimensions constituting KIDSCREEN.

**Methods:**

Students (*n* = 154) aged 15–16 years who were recruited from schools located in both low (two schools) and high (two schools) socioeconomic status (SES) areas in Sweden participated in a cross-sectional study. The adolescents answered a questionnaire including a new 6-item scale measuring perceived autonomy and HRQoL assessed by the KIDSCREEN-52 instrument. A factor analyses was computed to investigate the relation between the items in the AAC scale and the autonomy items in the KIDSCREEN instrument. Hierarchical regression analyses were computed to investigate if the AAC scale predicted HRQoL in any of the 10 dimensions in KIDSCREEN after controlling for gender, SES and the original autonomy scale included in KIDSCREEN.

**Results:**

The factor analysis showed that all the items from the autonomy scale loaded in one factor and that all the items from the AAC scale loaded in another dimension. The hierarchical regression models showed that the AAC scale uniquely predicted HRQoL in all dimensions of the KIDSCREEN instrument after controlling for gender, SES and the original autonomy scale included in KIDSCREEN-52. A high level of perceived autonomy was associated with a high level of HRQoL for both scales.

**Conclusion:**

A new scale for measuring autonomy from a control perspective has been presented and shown to differ from autonomy as volition. Both forms of autonomy are positively related to HRQoL in adolescence 15–16 years old.

**Supplementary Information:**

The online version contains supplementary material available at 10.1186/s12887-022-03607-5.

## Background

Health and well-being are essential in all human life. Health-related quality of life (HRQoL) is a multi-dimensional measure that relates to individuals’ perception and subjective evaluation of their health and well-being within their specific culture, following the intentions from WHO [1, 2]. Many measures of HRQoL concern special patient groups while others are generic, that is directed towards a general population [3]. The role of different aspects of autonomy for HRQoL among adolescents are however still poorly understood.

KIDSCREEN is a generic instrument measuring HRQoL in children and adolescents 8–18 years old. It was developed as an EU project including 13 European countries and was tested on over 3000 children. The instrument comes in 52, 27 and 10-item versions. All versions cover 10 dimensions: physical well-being, psychological well-being, moods and emotions, self-perception, parent relation and home life, financial resources, peers and social support, bullying, and autonomy [4]. Hence, autonomy is part of the very definition of the health-related quality of life of children and adolescents.

A close relation between autonomy and different measures of well-being is a common finding. Perceived autonomy in adolescence has been found to be negatively related to depression [5] and positively related to well-being [6]. Autonomy is also central to self-determination theory (SDT) in which autonomy, competence and relatedness constitute basic psychological needs and the satisfaction of these needs leads to increased well-being in both adolescents and adults [7], something that also has been verified in numerous empirical studies [8].

However, autonomy can be defined in many ways, for example as emotional independence, as volition versus pressure, functional independence and detachment, and its effect on health and well-being is therefore dependent on how it is defined [9]. Van Petegem and colleagues [9] investigated in two studies whether 8 and 14 scales related to autonomy could be reduced to a few dimensions. Indeed, the authors found that the different measures could be reduced to two approximately orthogonal dimensions labeled as “volition” and “distance and proximity”. Volition concern to what extent the adolescents experience they are acting from free will or coerced by someone else, and is influenced by SDT. Distance and proximity concern an emotional distance in the relations to the parents and is influenced by attachment theory [9]. While a high score on autonomy as volition was positively correlated to positive outcomes including well-being, a high score on autonomy as distance were negatively related to life-satisfaction and positively related to various problem behaviors [9]. Recently [10] it has become more common to distinguish between volition and independence where independence “is mainly about the question how much adolescents depend on others and who is regulating a certain behavior or goal (i.e., the parent, the adolescent or both)”. Volition and independence are often correlated, and it is a challenging research task to describe their interaction and under what conditions the outcome is beneficial [10].

The autonomy dimension in the KIDSCREEN instrument concern children’s opportunities to create social and leisure time [11] without further specification. Yet, it is quite clear from the items (i.e., *Do you have enough free time to be with your mates?*) that they concern autonomy as volition, and this is also supported by their positive relationship to the other dimensions in KIDSCREEN.

This conception of autonomy is compared to autonomy from a control perspective in the present study. Autonomy from this perspective emphasizes the importance of power and control for children’s development, in particular their experience of being seen and listened to and their opportunities to take part in decisions that concern themselves [12]. Yet, there is no available scale that directly address this aspect of autonomy in adolescence, although it is conceptually related to autonomy as independence [10]. To be clear, the concept of control is not new in relation to autonomy in adolescence, but it has been used to refer to parental control versus adolescence freedom of choice [13], and thus is akin to the volition-pressure dimension. The present measure concerns the adolescents’ sense of control and influence over things that happens in their lives.

The first aim of the present study was therefore to investigate the psychometric properties of a new scale measuring autonomy in adolescence from the control perspective (AAC) [12] including its relation to the autonomy dimension included in KIDSCREEN. A second aim was to investigate the relation between AAC and HRQoL. The second aim will be undertaken by investigating AACs ability to predict each of the 10 dimensions constituting KIDSCREEN-52 after controlling for extraneous variables.

## Methods

### Participants and procedure

A paper and pencil questionnaire was distributed to 154 students at four Swedish high schools during regular class time in a cross-sectional design. The questionnaire was filled out individually and was completed in approximately 20 min. The students attended grade 9 and were 15–16 years old. Two schools with 93 participating students (47 girls and 46 boys) were in low SES areas (mean income below the median) and two schools with 61 participating students (35 girls and 26 boys) were in high SES areas (mean income above the median). Principals and teachers at all four schools were contacted and all approved the study.

### Measures

HRQoL was measured using the KIDSCREEN-52 questionnaire [11], a cross-cultural measure of HRQoL that was developed and standardized in Europe. This instrument consists of 52 items measuring 10 HRQoL dimensions: physical well-being (5 items), psychological well-being (6 items), moods and emotions (7 items), self-perception (5 items), autonomy (5 items, henceforth labelled as KSA), parent relation and home life (6 items), social support and peers (6 items), school environment (6 items), social acceptance and bullying (3 items), and financial resources (3 items). Responses were given on two five-point Likert-type scales with alternatives ranging from ‘never’ to ‘always’ to assess frequency and from ‘not at all’ to ‘extremely’ to assess intensity. These scores were transformed according to recommendations from The KIDSCREEN Group Europe [11, 14] including computation of Rasch scores. Higher scores indicate a better HRQoL.

AAC was measured by a 6-item scale developed in the project to measure perceived possibilities for adolescents to influence their life. The scale contained the following questions (translated from Swedish): (1) *Do you find that adults don’t listen to you? (reversed)* (2) *Do you take part in important decisions concerning your life?* (3) *Do you find yourself treated unjustly because of your age? (reversed)* (4) *Do you feel that adults take you seriously?* (5) *Do you wish you could have a greater influence on your daily life?* (6) *Do you think your life would be better if more decisions were up to you?* Responses were given on a five-point Likert-type scale to each question with alternatives from 0 (‘never’) to 4 (‘always’). A high value indicated high autonomy.

### Data analysis

A principal component analysis (PCA) containing the 6 items from AAC and KSA was first computed determining the optimal number of dimensions and how much of the variance they explained. Next, a promax rotation was employed to find a distinct factor structure.

Nine hierarchical regression analyses were computed entering gender and SES in a first block, autonomy as measured by KIDSCREEN-52 in a second block, and autonomy measured by AAC in a third block. HRQoL in all nine dimensions except autonomy served as dependent variables. A two-block analysis with gender and SES in a first block and ACC in the second was employed for a tenth regression in which the autonomy dimension in KIDSCREEN-52 served as dependent variable. Level of significance was set to < 0.05.

## Results

Means (SD) for girls and boys in schools located in high and low SES areas for the AAC scale and all 10 dimensions of HRQoL from KIDSCREEN are shown in Table 1. A factor analysis was computed on all items measuring autonomy, that is, the 6 items constituting AAC and the 5 items constituting KSA. A scree plot indicated a two-dimensional solution explaining together 58% of the variance (36% + 22%). An oblique rotation using promax converged after 3 iterations and indicated a small correlation between the axes, *r*(153) = 0.205, *p* = 0.011. All five items from KSA loaded highest in the first dimension and all six items from AAC loaded highest in the second dimension (Table 2). The internal consistency yielded an alpha of 0.89 for KSA and 0.78 for AAC. The correlations for items within both scales were all significant (*p* < 0.01) although there were also some significant correlations between items across the scales (Table 3).

**Table 1:** Mean (SD) and Cronbach alpha of the Autonomy in Adolescence Control (AAC) scale and the 10 dimensions of Health-Related Quality of Life from the KIDSCREEN-52 instrument over SES, girls and boys (*N* = 154)

Dimension HRQoL	Cronbach alpha	Girls Means (SD)	Boys Means (SD)	High SES Means (SD)	Low SES Means (SD)	Total Means (SD)
AAC (6 items)	.78	21.06 (4.79)	21.47 (4.18)	21.28 (4.84)	21.24 (4.30)	21.25 (4.51)
Dimensions from KIDSCREEN-52						
Physical well-being (5 items)	.80	42.87 (7.86)	49.18 (13.42)	46.22 (10.05)	45.56 (12.00)	45.82 (11.24)
Psychological well-being (5 items)	.92	48.42 (12.26)	52.10 (12.34)	51.05 (9.84)	49.54 (13.83)	50.14 (12.39)
Moods and emotions (7 items)	.90	42.95 (13.85)	54.00 (11.89)	48.96 (14.76)	47.56 (13.64)	48.11 (14.06)
Self-perception (5 items)	.77	44.99 (11.57)	50.66 (11.65)	44.69 (10.45)	49.57 (12.47)	47.64 (11.92)
Autonomy (5 items)	.89	41.95 (13.34)	47.98 (12.45)	45.00 (10.76)	44.61 (14.69)	44.77 (13.24)
Parent relations and home life (6 items)	.90	52.81 (13.41)	54.47 (12.97)	50.99 (12.80)	55.29 (13.23)	53.59 (13.19)
Peers and social support (6 items)	.84	54.47 (10.51)	52.98 (15.30)	53.12 (14.57)	54.20 (11.83)	53.78 (12.95)
School environment (6 items)	.86	47.55 (11.24)	47.02 (12.46)	45.38 (9.62)	48.56 (12.92)	47.30 (11.79)
Social acceptance and bullying (4 items)	.78	52.84 (11.42)	49.11 (14.75)	54.60 (9.58)	48.80 (14.66)	51.10 (13.17)
Financial resources (3 items)	.93	53.57 (10.79)	55.56 (11.16)	54.19 (10.89)	54.71 (11.08)	54.50 (10.97)

*Note*: The values from the KIDSCREEN dimensions were transformed to Rasch scores

**Table 2:** Factor loadings from the KIDSCREEN autonomy items (KSA) and the items in the autonomy in adolescence control scale (AAC) (*N* = 154)

Item	Factor 1	Factor 2
KSA1	.780	.266
KSA2	.850	.104
KSA3	.814	.131
KSA4	.851	.145
KSA5	.847	.227
ACC 1	.235	.711
ACC 2	.281	.661
ACC 3	.165	.630
ACC 4	-.034	.747
ACC 5	.087	.623
ACC 6	.159	.749

*Note*: A principle component analysis with an promax rotation and Kaiser normalization was used

**Table 3:** Correlations between KIDSCREEN autonomy items (KSA) and items in the autonomy in adolescence control scale (AAC) (*N* = 154)

Items	KSA1	KSA2	KSA3	KSA4	KSA5	AAC1	AAC2	AAC3	AAC4	AAC5
KSA1:..have enough time for myself	1									
KSA2:..do what you want…	.587**	1								
KSA3:..opportunities to be outside.	.514**	.594**	1							
KSA4:..time to be with mates…	.581**	.612**	.705**	1						
KSA5:..choose what to do…	.601**	.742**	.555**	.623*	1					
AAC 1:..adult’s don’t listen to you	.168*	.172*	.126	.205*	.259**	1				
AAC 2:..take part in important decisions	.272**	.104	.247**	.209**	.208**	.363**	1			
AAC 3:..treated unjustly because of age	.165*	.124	.140	.111	.143	.611**	.297**	1		
AAC 4:..adults take you seriously	.268**	.038	.093	.094	.142	.226**	.588**	.290**	1	
AAC 5:..whish for greater influence.	.078	-.026	-.045	-.030	.042	.433**	.279**	.506**	.311**	1
AAC6:..better life if more decisions …	.113	.062	.053	.065	.151	.271**	.303**	.319**	.266**	.456**

*Note*: **p < .01, *p < .05

Next, a series of hierarchical regression analyses were computed in which gender and SES were inserted in a first block, KSA in a second block and ACC in a third block. Each dimension of HRQoL as measured by KIDSCREEN-52 served as dependent variable. An exception was the autonomy dimension of KIDSCREN for which the second block (KSA) was omitted. Collinearity statistics revealed that tolerance for all analyses were above 0.9 and VIF were below 1.1. Hence, multicollinearity was no problem.

The regression analyses showed that gender and SES (block 1) accounted together for between 1–16% of the variance in each dimension of HRQoL. They contributed significantly to *Physical well-being* (8%), *Moods and emotions* (16%), *Self-perception* (9%), *Autonomy* (5%), and *Social acceptance and bullying* (6%) (Table 4)*.*

**Table 4:** Hierarchical multiple regression analyses showing the unique contribution of the Autonomy in Adolescence Control (AAC) scale entered in the final block after correction for gender (girls = 0, boys = 1), SES (0 = low, 1 = high) and the total autonomy dimension from KIDSCREEN (KSA) when applicable predicting all 10 dimensions in the KIDSCREEN – 52 Instrument (*N* = 154)

**Model**	***B***	***SE*** ***B***	***β***	***Δ*** ***R***^**2**^	**Model** ***R***^**2(adj)**^
Physical well-being					.31***
Block 1 (Gender, SES)				.08**	
Block 2 (KSA)				.20***	
Block 3				.045**	
Gender	3.99	1.55	.178*		
SES	.77	1.55	.034		
KSA	.36	.06	.42***		
AAC	.54	.17	.22**		
Psychological well-being					.25***
Block 1 (Gender, SES)				.03	
Block 2 (KSA)				.19***	
Block 3				.05**	
Gender	1.23	1.78	.05		
SES	1.43	1.77	.06		
KSA	.38	.07	.40***		
AAC	.65	.20	.24**		
Moods and emotions					.40***
Block 1 (Gender, SES)				.16***	
Block 2 (KSA)				.11***	
Block 3				.14***	
Gender	8.98	1.82	.32***		
SES	1.86	1.81	.07		
KSA	.28	.07	.27***		
AAC	1.20	..200	.39***		
Self-perception					.33***
Block 1 (Gender, SES)				.09**	
Block 2 (KSA)				.10***	
Block 3				.16***	
Gender	3.61	1.63	.15*		
SES	-4.76	1.62	-.20**		
KSA	.21	.06	.24**		
AAC	1.07	.18	.41***		
Autonomy					.09*
Block 1 (Gender, SES)				.05*	
Block 2 (AAC)				.03*	
Gender	5.86	2.07	.22**		
SES	.77	2.11	.03		
AAC	.53	.23	.18*		
Parent relations and home life					.32***
Block 1 (Gender, SES)				.03	
Block 2 (KSA)				.16***	
Block 3				.15***	
Gender	-1.15	1.81	-.04		
SES	-4.55	1.80	-.17*		
KSA	.34	.07	.34***		
AAC	1.15	.20	.39***		
Peers and social support					.07**
Block 1 (Gender, SES)				.01	
Block 2 (KSA)				.04**	
Block 3				.05**	
Gender	-2.85	2.08	-.11		
SES	-1.37	2.06	-.05		
KSA	.17	.08	.17*		
AAC	.63	.23	.22**		
School environment					.24***
Block 1 (Gender, SES)				.02	
Block 2 (KSA)				.19***	
Block 3				.05**	
Gender	-3.19	1.72	-.14		
SES	-3.56	1.70	-.15*		
KSA	.36	.07	.41***		
AAC	.56	.19	.22**		
Social acceptance and bullying					.13***
Block 1 (Gender, SES)				.06**	
Block 2 (KSA)				.00	
Block 3				.09***	
Gender	-3.39	2.04	-.13		
SES	5.56	2.03	.21**		
KSA	-.06	.08	-.06		
AAC	.90	.22	.31***		
Financial resources					.17***
Block 1 (Gender, SES)				.01	
Block 2 (KSA)				.05**	
Block 3				.14***	
Gender	.79	1.66	.04		
SES	-.56	1.17	-.03		
KSA	.13	.06	.16**		
AAC	.91	.18	.37***		

*Note*: Regression coefficients are reported for the final model. * *p* < .05; ** *p* < .01; *** *p* < .001

*KSA* (block 2) accounted for between 0–20% of the variance in addition to gender and SES (*Δ R*^***2***^*)*. *KSA* contributed significantly to all HRQoL dimensions of the KIDSCREEN-52 instrument except to *Social acceptance and bullying* (Table 4), and was particularly high for *Physical well-being* (20%), *Psychological well-being* (19%), *Moods and emotions* (11%), *Self-perception* (10%), *Parent relations and home life* (16%), and *School environment* (19%).

AAC (block 3) accounted for between 3–16% of the variance in addition to gender, SES and KSA. High AAC contributed significantly to all HRQoL dimensions of the KIDSCREEN-52 instrument including *Autonomy* (for which only two blocks were computed). Although the unique contribution of AAC was significant for all dimensions, the contribution was small in half of the cases (5% or less). However, the contributions to the explained variance for *Moods and emotions* (14%), *Self-perception* (16%), *Parent relations and home life* (15%), *Social acceptance and bullying* (9%), and *Financial resources* (14%) were substantial. See Table 4. In agreement with the low correlation between the two dimensions of autonomy, volition versus control, the correlation between the explained variance by KSA and AAC was small, (*r*(8) = 0.038, *p* = 0.92). Hence, their relation is neither antagonistic nor concurrent.

Results from the full models including the third block (2^nd^ for *Autonomy*) in the regressions revealed that gender predicted HRQoL relative to *Physical well-being, Moods and emotions, Self-perception*, and *Autonomy*. Boys reported significantly higher HRQoL than girls in all four of the above dimensions. SES predicted HRQoL relative to *Self-perception, Parent relations and home life*, *School environment*, and *Social acceptance and bullying*. Adolescents from low SES district schools reported significantly higher HRQoL than adolescents from high SES schools for *Self-perception, Parent relations and home life*, and *School environment,* whereas the pattern was reversed for HRQoL related to and *Social acceptance and bullying* (Table 4).

## Discussion

The present study has shown that AAC, a new scale measuring autonomy in adolescence, based on their sense of control [12] has sound psychometric properties and is different from a measure of autonomy based on volition and pressure as expressed in the autonomy part of KIDSCREEN-52, here named KSA. Moreover, AAC predicted HRQoL in all 10 dimensions of the KIDSCREEN-52 instrument in addition to gender, SES and KSA. AAC accounted uniquely for 3–16% of the variance in HRQoL. The contribution from ACC was always positive, the more autonomy in the control sense experienced by the adolescence, the higher was their HRQoL. This finding supports earlier claims that autonomy is important for our well-being [5–8] and extend it by showing that it is not only true for autonomy as volition but also for autonomy in the control sense, for example experiencing influence over what happens in the adolescent’s life [12].

In agreement with previous studies, we found that HRQoL was higher in 15–16-year-old boys as compared with same aged girls [14–16], and that HRQoL was affected by SES [17–19]. However, whereas other studies on SES have reported that adolescents from high SES families consistently have higher HRQoL, only one dimension (*social acceptance and bullying)* in the present study was in this direction. For the other three dimensions with significant contributions from SES (*self-perception, parent relations and home life* and *school environment),* low SES adolescents reported the highest HRQoL. One reason for this difference might be that SES in the present study was measured indirectly by school location and not on the individual level. This might have weakened the predictive power of SES. Yet, effects of gender and SES were included in the present study for the purpose of control and the sample size was too small to investigate their effect on HRQoL more thoroughly.

The small sample size was a major limitation to the main results of this study. However, the relation between AAC and a multidimensional measure of well-being such as KIDSCREEN-52 was a major strength of the present study, demonstrating its associations to many areas in life. It is plausible that the control aspect of autonomy is more important for adolescents than for younger children. Inclusion of younger age groups and ideally longitudinal studies are therefore needed in future studies.

To give a young person power and control over her or his life should not be confused with parental neglect. A multitude of sources has shown that parental neglect has aversive effects on children’s development [20]. However, a high score on the ACC scale indicate that parents care about their children. They listen to them and treat them justly and seriously. This is quite different from parent neglect. Yet, an empirical corroboration of this assumption would be valuable.

The present research also raises the question of whether HRQoL measures such as KIDSCREEN would be improved if more aspects than volition was measured in the autonomy dimension. This is a challenging thought but must stay as such until some of the limitations of the present study on AAC has been overcome. The KIDSCREEN group might also have some ideas about the relative contribution to HRQoL from each of the included dimensions.

## Conclusions

Autonomy is a multifaceted concept and has been measured in many ways. A scale for measuring adolescent’s sense of control and influence over things that happen in their lives has been presented and shown to differ from autonomy as volition in adolescence 15–16 years old. Both forms of autonomy are positively related to HRQoL in adolescence.

## Supplementary Information

**Additional file 1.**

## Data Availability

The data sets used and analyzed during the current study are available from the corresponding author on request.
